# How do social and gender norms influence the sexual and reproductive health of younger adolescents in sub-Saharan Africa? A scoping review protocol

**DOI:** 10.12688/wellcomeopenres.23139.1

**Published:** 2024-11-12

**Authors:** Fardawsa Ahmed, Owen Nyamwanza, Jermaine Dambi, Frances Cowan, Webster Mavhu

**Affiliations:** 1 Department of International Public Health, Liverpool School of Tropical Medicine, Liverpool, England, UK; 2 SRH & MNCH, Center for Sexual Health and HIV/AIDS Research (CeSHHAR), Harare, Zimbabwe; 3 Rehabilitation Sciences Unit, Faculty of Medicine and Health Sciences, University of Zimbabwe, Harare, Zimbabwe

**Keywords:** Scoping review protocol, Social and gender norms, Very young adolescents, Sexual and reproductive health, Sub-Saharan Africa

## Abstract

**Introduction:**

Younger adolescents (aged 10–14 years) in sub-Saharan Africa (SSA) have disproportionate sexual and reproductive health (SRH) outcomes due to structural, behavioural, socioeconomic and other factors. Social and gender norms have important consequences for the SRH and wellbeing of younger adolescents both now and over their life course. SRH programming often focuses on older adolescents (aged 15–19 years), overlooking younger ones. This scoping review sets out to explore how gender and social norms influence younger adolescents’ SRH in SSA, to inform tailored interventions.

**Methods:**

The Arksey and O'Malley strategy will be used to review the available literature. Online databases (PubMed/MEDLINE, CINHAL, EMBASE, PsycINFO, Cochrane Library, and African Index Medicus) will be searched for original studies published between 1 January 2000 and 30 September 2024. Further, a manual search to include relevant grey literature will be performed. The steps in the review are: 1) defining the research question, 2) identifying relevant studies, 3) selecting studies, 4) charting the data, and 5) collating, summarising, and reporting the results.

**Results:**

Findings will be reported in accordance with the guidance provided in the Preferred Reporting Items for Systematic Reviews and Meta-Analyses Protocols (PRISMA-P) statement.

**Discussion:**

The review will generate the most up-to-date evidence and identify gaps in literature in addition to informing future research on how gender and social norms influence younger adolescents’ SRH in SSA. Findings will inform and influence future interventions and evaluations in this area.

## Plain language summary

This study protocol outlines a scoping review to explore how social and gender norms influence younger adolescents' sexual and reproductive health in sub-Saharan Africa. The protocol follows the Arksey and O'Malley strategy to review the available literature. The key steps to be followed in the review are: defining the research question, identifying relevant studies, selecting studies, charting the data, and collating, summarising, and reporting the results. The review aims to inform future research on how gender and social norms influence younger adolescents’ sexual and reproductive health in addition to informing and influencing interventions and evaluations in this area.

## Introduction

Adolescence is a critical life stage as it signifies the shift from childhood to physical, psychological and social maturity ^1^. During this phase, adolescents develop and build knowledge and abilities to address crucial aspects of their health and development while their bodies undergo maturation ^2^. There are 1.3 billion adolescents worldwide, accounting for 16% of the global population ^3^. About half are classified as younger adolescents (aged 10–14 years), of whom around 90% reside in low- and middle-income countries ^4^. In sub-Saharan Africa (SSA), adolescents are a particularly important group as the number of young people in Africa is projected to double in the next 30 years ^5^.

The World Bank predicts that Africa's ability to benefit from the projected population growth directly depends on the health and well-being of today’s adolescents ^5^. Therefore, it is crucial to invest in the health of adolescents across a wide range of outcomes, which can lead to broader societal gains, including improved productivity and economic gains. Focusing on sexual and reproductive health (SRH) is critical given adolescents in SSA have the worst SRH outcomes of this age group worldwide ^6, 7^. For example, unmarried girls in SSA have the highest rates of abortion and abortion-related morbidity and mortality of any region ^6, 7^. Rates of curable sexually transmitted infections are higher, and key SRH service indicators such as coverage of antenatal syphilis screening are poorer than in any other World Health Organisation (WHO) region ^8^. Further, adolescent girls in the SSA account for 75% of global new HIV infections ^9^. These SRH challenges are due to a range of factors - structural, behavioural, socioeconomic and sociocultural ^10, 11^.

Social norms are unwritten or informal social rules that determine how people ought to behave in certain situations ^12^. Gender norms are a subtype of social norms that dictate how men and women should behave ^13^. Social and gender norms are both accompanied by sets of positive and negative sanctions for norm abiders/adherents and violators alike ^14^. Relatedly, social and gender norms are hinged on, and reflect, the predominantly patriarchal character of most SSA societies ^15^. Patriarchy, the social and ideological classification of men as superior and women as subordinate and dependent, is an essential determinant of gender relations ^16^. Patriarchal structures confer power on men to control resources and dominate women, leading to social and gender norms that are favourable to the former and punitive to the latter ^17^.

While gender socialisation begins in childhood, it intensifies in early adolescence (10–14 years) and solidifies in later adolescence (≥15 years) ^18– 20^. However, the plasticity of the early adolescent brain offers a prime opportunity to shape self-perception and behaviour and, manipulate social constructs ^19^. Intervening in early adolescence, when attitudes and behaviours are still malleable, provides the opportunity to promote gender-equitable identities and challenge inequitable gender stereotypes before they are solidified and become less amenable to change. Once positive social and gender norms are inculcated, their impact has important consequences for the SRH and wellbeing of adolescents both now and over their life course ^21^.

In SSA, SRH programmes have mostly focused on older adolescents (aged 15–19 years), partly due to the existence of nationally representative data obtained through Demographic and Health Surveys ^4^. These surveys typically gather SRH data from those aged ≥15 years. Resultantly, there is a paucity of research on the influence of gender and social norms on younger adolescents’ SRH. The proposed scoping review seeks to address this gap by identifying and analysing literature on how social and gender norms influence younger adolescents’ SRH in SSA. Such information is essential for the development of bespoke interventions, including informing SRH programming in SSA.

## Methods

We will employ the scoping review framework developed by Arksey and O’Malley ^22^. The review stages include: 1) defining the research question, 2) identifying relevant studies, 3) selecting studies, 4) charting the data, and 5) collating, summarising and reporting the results ^22^.

### Stage 1: Defining the research question

The review research questions are informed by the Population–Concept–Context (PCC) framework ^23^. The main review question is *"How do social and gender norms influence the SRH of younger adolescents (aged 10–14) in SSA?"* A sub-question is *"What gaps exist in interventions relating to social and gender norms and SRH of younger adolescents in SSA?"*. By addressing these questions, we will be able to outline the range of relevant literature around these aspects and inform the direction of future initiatives.

### Stage 2: Identifying relevant studies

We will develop a comprehensive search strategy to identify relevant studies written in English from 1 January 2000 to 30 September 2024. We do not have resources to analyse studies written in other languages, and we recognise that this is a potential limitation of our review. We chose 2000 as our baseline year as this is about when research on social and gender norms, younger adolescents, and SRH issues in SSA intensified, especially as part of the Millennium/Sustainable Development Goals' global health programmes ^24^. For example, Sustainable Development Goal (SDG) 5 recognises gender equality as a fundamental human right and a necessary foundation for a peaceful, prosperous and sustainable world ^25^.

We will search PubMed/MEDLINE, CINHAL, EMBASE, PsycINFO, Cochrane Library, and African Index Medicus to identify the relevant peer-reviewed studies. We will also search websites of organisations focusing on adolescent SRH, including, WHO, UNICEF, and The International Association of Adolescent Health (IAAH). We will conduct a multi-stage, iterative literature search in the afore-mentioned electronic databases. For instance, we will apply a combination of Boolean logic operators to glean the literature in PubMed. The first search will include comprehensive data collection utilising MeSH keywords or specific search phrases, as shown in Table 1.

**Table 1.** Search strategy.

Concept	Search Terms
Social norms	“Social norms” OR “Cultural pluralism” OR “Social Attitudes” OR “sociocultural restrictions” OR “protective behaviours” OR “African culture”
Sexual and reproductive health	“Sexual behaviour” OR “sexual health” OR “Youth sexual behaviour” OR “Attitudes toward sex” OR “Sexuality” OR “Sexual Health -- In Adolescence” OR “Reproductive Health -- In Adolescence” OR “contraceptives” OR “family planning” OR “Protected sex” OR “HIV” OR “STI” OR “sexual transmitted diseases”
Younger adolescents	“Youth” OR “young person” OR “minor” OR “10-14 years old” OR “young adolescent*” OR “Very young adolescents” OR “early adolescent”
Sub-Saharan Africa	“Angola” OR “Benin” OR “Botswana” OR “Burkina Faso” OR “Burundi” OR “Cameroon” OR “Cape Verde” OR “Central African Republic” OR “CHAD” OR “Comoros” OR “Congo” OR “Congo Democratic Republic” OR “Djibouti” OR “Equatorial Guinea” OR “Eritrea” OR “Ethiopia” OR “Gabon” OR “Gambia” OR “Ghana” OR “Guinea” OR “Guinea-Bissau” OR “Cote d'Ivoire” OR “Ivory Coast” OR “Kenya” OR “Lesotho” OR “Liberia” OR “Madagascar” OR “Malawi” OR “Mali” OR “Mozambique” OR “Namibia” OR “Niger” OR “Nigeria” OR “Sao Tome and Principe” OR “Rwanda” OR “Senegal” OR “Seychelles” OR “Sierra Leone” OR “Somalia” OR “South Africa” OR “South Sudan” OR “Sudan” OR “Swaziland” OR “Tanzania” OR “Togo” OR “Uganda” OR “Zambia” OR “Zimbabwe” OR “Africa, South of the Sahara” OR “sub-Saharan Africa''

To narrow this search further, we will add more MeSH or search terms, including "culture", "cultural norms/practices/beliefs/experiences/factors", "community”, "indigenous/traditional African care practices/systems" to the first broad catch. As indicated in the inclusion and exclusion criteria, the search will have geographical, age and time limitations. Moreover, we will explore the reference lists of included articles to find additional relevant studies. Further, grey literature, including reports and policy documents, will be searched and included where necessary.

### Stage 3: Study selection

***Inclusion/exclusion criteria*.** Guided by the PCC framework ^23^, we will include studies meeting at least one of the criteria in each category outlined in Table 2. We will include studies that employed quantitative, qualitative, or mixed methods. We will exclude reviews (scoping, narrative, systematic, meta-analyses, etc.), personal opinion articles, and conceptual or theoretical articles that neither analyse primary nor secondary data. We will also exclude non-English texts. We will account for all excluded material to appreciate any potential biases or implications of the exclusions to our findings.

**Table 2.** Inclusion (PCC) framework.

Framework item	Item components
Population(s)	-Younger adolescents, both female and male (aged 10–14 years)
Concept(s)	-Gender norms -Social norms -Sexual and reproductive health
Context(s)	-Studies conducted in SSA between 1 January 2000 and 30 September 2024 (inclusive)

***Search outcomes*.** We will perform first-level de-duplication in EndNote and second-level de-duplication will be done in Covidence. Two independent reviewers (FA & ON) will review all the included and excluded papers to avoid selection bias.

***Quality appraisal*.** We will assess quality using relevant tools and approaches depending on a study’s design and methodology.

### Stage 4: Data charting

Data charting refers to the process of synthesising and interpreting data through sorting, charting, and organising information based on key themes and issues ^22^. In line with Levac *et al.*’s ^26^ recommendations, we will develop a standard Excel data charting form and populate it with key study characteristics (e.g., author(s), publication year, study title, geographical region [urban/rural], study design, study methods, and key themes and issues from each selected study vis-à-vis the review objectives. This will be an iterative process, involving back-and-forth data extraction and subsequent updating of the data charting form.

We will begin the data charting process with a pilot phase where two independent reviewers (FA & ON) will chart data from a random sample of 5–10 of the final selected studies. This piloting process will determine whether or not both reviewers’ independent chartings align with the review objectives, and allow for any changes to the data charting form. Any disagreements will be discussed with an expert (WM), and the data charting form will be revised accordingly.

## Results

### Stage 5: Collating summarising and reporting results

Following Levac *et al.*’s ^26^ suggestions, we will synthesise our data in three phases. Firstly, we will analyse and summarise all charted data about study characteristics using descriptive statistics. We will use thematic analysis to analyse and summarise all charted data relating to key themes and issues vis-à-vis the review objectives. We will present quantitative results using tables and/or graphs and qualitative findings using key themes. Secondly, we will conduct our reporting iteratively and continuously adjust our reporting scheme. Finally, we will discuss the meanings of our findings, including implications for policy, practice and further research.

## Discussion

Knowledge around influence of social and gender norms on SSA’s younger adolescents’ SRH is currently limited ^10^. This scoping review will synthesise current literature to address this critical knowledge gap. By mapping the breadth of studies focusing on social and gender norms, and SRH among younger adolescents, the scoping review will provide a critical evidence base to inform the development and implementation of culturally relevant interventions and further research to help improve adolescents’ SRH outcomes in SSA. Specifically, review findings will inform the co-development and evaluation of a peer-delivered gender-transformative intervention for in- and out-of-school younger adolescents to promote positive masculinity and sexual health in Zimbabwe. The ultimate goal is to develop an effective model for scale-up to influence constructs of positive masculinity.

We will share review results with diverse stakeholders through various channels including peer-reviewed publications, conferences, stakeholder meetings with organisations working with (younger) adolescents and other partners implementing programmes that seek to address SRH outcomes among adolescents in SSA. We aim to inform and influence future interventions and evaluations in this area.

## Conclusion

Here, we present a protocol to scope existing research to understand social and gender norms and their influence on younger adolescents’ SRH in SSA. The scoping review will contribute to the existing literature by addressing knowledge gaps on these aspects. The review will provide valuable insights for the development of tailored interventions for this important group.

## Ethics and consent

Ethical approval and consent were not required.

## Data Availability

No data are associated with this article. Preferred Reporting Items for Systematic Reviews and Meta-Analyses Protocols (PRISMA-P) checklist is available on Open Science Framework repository. Open Science Framework: PRISMA-P checklist for How do social and gender norms influence the sexual and reproductive health of younger adolescents in sub-Saharan Africa? A scoping review **DOI** – 10.17605/OSF.IO/EW7S5 ^27^. Data are available under the terms of the Creative Commons Zero "No rights reserved" data waiver (CC0 1.0 Public domain dedication).
