# How do social norms influence the sexual and reproductive health of very young adolescents in sub-Saharan Africa? A scoping review protocol

**DOI:** 10.12688/wellcomeopenres.23139.2

**Published:** 2025-02-28

**Authors:** Fardawsa Ahmed, Owen Nyamwanza, Alice N. Ladur, Jermaine Dambi, Frances Cowan, Webster Mavhu

**Affiliations:** 1 Department of International Public Health, Liverpool School of Tropical Medicine, Liverpool, England, UK; 2 SRH & MNCH, Center for Sexual Health and HIV/AIDS Research (CeSHHAR), Harare, Zimbabwe; 3 Rehabilitation Sciences Unit, Faculty of Medicine and Health Sciences, University of Zimbabwe, Harare, Zimbabwe

**Keywords:** Scoping review protocol, Social and gender norms, Very young adolescents, Sexual and reproductive health, Sub-Saharan Africa

## Abstract

**Introduction:**

Introduction Very young adolescents (VYAs, aged 10–14 years) in sub-Saharan Africa (SSA) have the worst sexual and reproductive health (SRH) outcomes of this age group worldwide due to structural, behavioural, socioeconomic and other factors. Social and gender norms have important consequences for the SRH and wellbeing of VYAs both now and over their life course. SRH programming often focuses on older adolescents (aged 15–19 years), overlooking younger ones. This scoping review sets out to explore how social and gender norms influence VYAs’ SRH in SSA, in addition to exploring interventions that have been effective, to inform a context-specific intervention.

**Methods:**

We will employ the methodology developed by Arksey and O'Malley to review the available literature. We will search online databases (PubMed/MEDLINE, CINHAL, EMBASE, PsycINFO, Cochrane Library, and African Index Medicus) for original studies published between 1 January 2000 and 31 December 2024. Further, we will perform a manual search to include relevant grey literature. The steps in the review are: 1) defining the research question, 2) identifying relevant studies, 3) selecting studies, 4) charting the data, and 5) collating, summarising, and reporting the results.

**Results:**

Results We will report findings in accordance with the guidance provided in the Preferred Reporting Items for Systematic Reviews and Meta-Analyses Protocols (PRISMA-P) statement. We will analyse and summarise data about study characteristics using descriptive statistics. We will use thematic analysis to analyse and summarise key themes and issues. We will triangulate quantitative and qualitative findings.

**Discussion:**

The review will map the breadth of studies focusing on social and gender norms, and SRH among VYAs, in addition to exploring interventions that have been effective. Findings will help us and others develop context-specific, bespoke interventions.

## Plain language summary

This study protocol outlines a scoping review to explore how social and gender norms influence younger adolescents' sexual and reproductive health in sub-Saharan Africa. The protocol follows the Arksey and O'Malley strategy to review the available literature. The key steps to be followed in the review are: defining the research question, identifying relevant studies, selecting studies, charting the data, and collating, summarising, and reporting the results. The review aims to inform future research on how gender and social norms influence younger adolescents’ sexual and reproductive health in addition to informing and influencing interventions and evaluations in this area.

## Introduction

Adolescence is a critical life stage as it signifies the shift from childhood to physical, psychological and social maturity ^1^. During this phase, adolescents develop and build knowledge and abilities to address crucial aspects of their health and development while their bodies undergo maturation ^2^. Exactly when adolescence starts and ends has been a subject of debate, especially as some view a 10-year-old as a child and a 19-year-old as an adult ^2^. We are guided by the World Health Organisation (WHO) which defines “adolescents” as individuals in the 10–19 years age group, “youth” as those in the 15–24 years range, while “young people” covers those 10–24 years old ^3^.

There are ∼1.3 billion adolescents (10–19 years old) worldwide, accounting for 16% of the global population ^1^. About half are classified as very young adolescents (VYAs aged 10–14 years), of whom around 90% reside in low- and middle-income countries ^4^. In sub-Saharan Africa (SSA), adolescents are a particularly important group as the number of young people in Africa is projected to double in the next 30 years ^5^.

The World Bank predicts that Africa's ability to benefit from the projected population growth directly depends on the health and well-being of today’s adolescents ^5^. Therefore, it is crucial to invest in the health of adolescents across a wide range of outcomes, which can lead to broader societal gains, including improved productivity and economic gains. Focusing on sexual and reproductive health (SRH) is critical given adolescents in SSA have the worst SRH outcomes of this age group worldwide ^6, 7^. For example, unmarried girls in SSA have the highest rates of abortion and abortion-related morbidity and mortality of any region ^6, 7^. Rates of curable sexually transmitted infections are higher, and key SRH service indicators such as contraceptive use, antenatal visits/adherence or HIV testing are poorer than in any other World Health Organisation (WHO) region ^8, 9^. Further, adolescent girls in SSA account for 75% of new HIV infections globally ^10^. These SRH challenges are due to a range of factors - structural, behavioural, socioeconomic and sociocultural, including social norms ^11, 12^.

Social norms are unwritten or informal social rules that determine how people ought to behave in certain situations ^13^. Gender norms are a subtype of social norms that dictate how men and women should behave ^13^. Social and gender norms are both accompanied by sets of positive and negative sanctions for norm abiders/adherents and violators alike ^14^. Relatedly, social and gender norms are hinged on, and reflect, the predominantly patriarchal character of most SSA societies ^15^. Patriarchy, the social and ideological classification of men as superior and women as subordinate and dependent, is an essential determinant of gender relations ^16^. Patriarchal structures confer power on men to control resources and dominate women, leading to social and gender norms that are favourable to the former and punitive to the latter ^17^.

There are several other intertwined norms related to virginity, control of female sexuality, fertility and childbearing, and family planning use ^15, 18, 19^. These either facilitate or impede healthy SRH behaviours and service seeking. For example, females who either purchase condoms or suggest their use are considered ‘loose’ and therefore, both unfit and unsuitable for marriage ^15, 18, 19^. Such negative “sanctions” contribute to the poor SRH outcomes described earlier.

While gender socialisation begins in childhood, it intensifies in early adolescence (10–14 years) and solidifies in later adolescence (≥15 years) ^20– 22^. However, the plasticity of the early adolescent brain offers a prime opportunity to shape self-perception and behaviour and, manipulate social constructs ^21^. Intervening in early adolescence, when attitudes and behaviours are still malleable, provides the opportunity to promote gender-equitable identities and challenge inequitable gender stereotypes before they are solidified and become less amenable to change. Once positive social and gender norms are inculcated, their impact has important consequences for the SRH and wellbeing of adolescents both now and over their life course ^13^.

In SSA, SRH programmes have mostly focused on older adolescents (aged 15–19 years) (aged 15–19 years) ^23^. This is due to at least two reasons. Firstly, early adolescence (10–14 years) is generally a time of good health, when this group is not as vulnerable to illness as when they are younger ^24^. Secondly there are (mistaken) beliefs that VYAs are too young to be provided with information on SRH and/or that they are not sexually active ^25– 27^, which serve as obstacles to both research and program development. Resultantly, there are fewer initiatives focusing on VYAs ^28^. An exception is the Global Early Adolescent Study (GEAS) ^28^.

The GEAS is a longitudinal, multi-country initiative that is exploring VYAs’ perceptions of the gender norms that regulate their behaviour, how they form their own beliefs about gender, and how these beliefs align with social norms in their communities, including in four African countries: Democratic Republic of Congo, Kenya, Malawi and South Africa ^29^. An important observation has been that, as with other population groups, intervention effects do differ by context, and results can be highly contextual, even for settings generally considered ‘similar’ ^30^. Indeed, there is increased recognition that African masculinities are produced in unique and varying contexts of intersections (including class, ethnicity, sexuality) ^31^. This points to the need for context-specific, bespoke initiatives.

The proposed review seeks tomap the breadth of studies focusing on social and gender norms, and SRH among VYAs, in addition to exploring interventions that have been effective. Findings will inform the development of a context-specific, bespoke intervention

## Methods

We will employ the scoping review framework developed by Arksey and O’Malley ^32^. The review stages include: 1) defining the research question, 2) identifying relevant studies, 3) selecting studies, 4) charting the data, and 5) collating, summarising and reporting the results ^32^.

### Stage 1: Defining the research question

The review research questions are informed by the Population–Concept–Context (PCC) framework ^33^. The main review question is *"How do social and gender norms influence the SRH of very young adolescents (aged 10–14) in SSA?"* A sub-question is *"What interventions relating to social and gender norms and SRH of VYAs in SSA have been effective?"*. By addressing these questions, we will be able to outline the range of relevant literature around these aspects and inform the development of a context-specific, bespoke intervention.

### Stage 2: Identifying relevant studies

We will develop a comprehensive search strategy to identify relevant studies written in English from 1 January 2000 to 31 December 2024. We chose 2000 as our baseline year as this is about when research on social and gender norms, VYAs, and SRH issues in SSA intensified, especially as part of the Millennium/Sustainable Development Goals' global health programmes ^34^. For example, Sustainable Development Goal (SDG) 5 recognises gender equality as a fundamental human right and a necessary foundation for a peaceful, prosperous and sustainable world ^35^.

We will search PubMed/MEDLINE, CINHAL, EMBASE, PsycINFO, Cochrane Library, and African Index Medicus to identify the relevant peer-reviewed studies. We will also search websites of organisations focusing on adolescent SRH, including, WHO, UNICEF, and The International Association of Adolescent Health (IAAH). We will conduct a multi-stage, iterative literature search in the afore-mentioned electronic databases. For instance, we will apply a combination of Boolean logic operators to glean the literature in PubMed. The first search will include comprehensive data collection utilising MeSH keywords or specific search phrases, as shown in Table 1.

**Table 1.** Search strategy.

Concept	Search Terms
Social norms	“Social norms” OR “Cultural pluralism” OR “Social norms attitudes” OR “Social norms perceptions” OR “sociocultural restrictions” OR “protective behaviours” OR “African culture”
Gender norms	“Gender” OR “Gender norms” OR “Gender norms attitudes” OR “Gender norms perceptions” OR “Gender roles” OR “Gender attitudes” OR “Gender practices” OR “Gender Transform*”
Sexual and reproductive health	“Sexual behaviour” OR “sexual health” OR “Youth sexual behaviour” OR “Attitudes toward sex” OR “Sexuality” OR “Sexual Health -- In Adolescence” OR “Reproductive Health -- In Adolescence” OR “contraceptives” OR “family planning” OR “Protected sex” OR “HIV” OR “STI” OR “sexual transmitted diseases” OR “pregnancy”
Younger adolescents	“Youth” OR “young person” OR “minor” OR “10-14 years old” OR “adolescent” OR “teenage” OR “young adolescent*” OR “Very young adolescents” OR “early adolescent”
Sub-Saharan Africa	“Angola” OR “Benin” OR “Botswana” OR “Burkina Faso” OR “Burundi” OR “Cameroon” OR “Cape Verde” OR “Central African Republic” OR “CHAD” OR “Comoros” OR “Congo” OR “Congo Democratic Republic” OR “Djibouti” OR “Equatorial Guinea” OR “Eritrea” OR “Ethiopia” OR “Gabon” OR “Gambia” OR “Ghana” OR “Guinea” OR “Guinea-Bissau” OR “Cote d'Ivoire” OR “Ivory Coast” OR “Kenya” OR “Lesotho” OR “Liberia” OR “Madagascar” OR “Malawi” OR “Mali” OR “Mozambique” OR “Namibia” OR “Niger” OR “Nigeria” OR “Sao Tome and Principe” OR “Rwanda” OR “Senegal” OR “Seychelles” OR “Sierra Leone” OR “Somalia” OR “South Africa” OR “South Sudan” OR “Sudan” OR “Swaziland” OR “Tanzania” OR “Togo” OR “Uganda” OR “Zambia” OR “Zimbabwe” OR “Africa, South of the Sahara” OR “sub-Saharan Africa''

To narrow this search further, we will add more MeSH or search terms, including "culture", "cultural norms/practices/beliefs/experiences/factors", "community”, "indigenous/traditional African care practices/systems" to the first broad catch. As indicated in the inclusion and exclusion criteria, the search will have geographical, age and time limitations. Moreover, we will explore the reference lists of included articles to find additional relevant studies. Further, grey literature, including reports and policy documents, will be searched and included where necessary.

### Stage 3: Study selection

***Inclusion/exclusion criteria***. Guided by the PCC framework ^33^, we will include studies focusing on VYAs, social norms/attitudes or perceptions of social norms (including gender norms) and SRH and, conducted in SSA between 1 January 2000 and 31 December 2024 (inclusive) ( Table 2). We will include studies that employed quantitative, qualitative, or mixed methods. We will exclude reviews (scoping, narrative, systematic, meta-analyses, etc.), personal opinion articles, and conceptual or theoretical articles that neither analyse primary nor secondary data. We will also exclude non-English texts. We will account for all excluded material to appreciate any potential biases or implications of the exclusions to our findings.

**Table 2.** Inclusion (PCC) framework.

Framework item	Item components
Population(s)	-Very young adolescents, both female and male (aged 10–14 years)
Concept(s)	-Social norms/attitudes or perceptions of social norms: -Gender norms/attitudes or perceptions of gender norms: -Sexual and reproductive health
Context(s)	-Studies conducted in SSA between 1 January 2000 and 31 December 2024 (inclusive)

***Search outcomes***. We will perform first-level de-duplication in EndNote and second-level de-duplication will be done in Covidence. Two independent reviewers (FA & ON) will review all the included and excluded papers to avoid selection bias.

### Stage 4: Data charting

Data charting refers to the process of synthesising and interpreting data through sorting, charting, and organising information based on key themes and issues ^32^. In line with Levac *et al.*’s ^36^ recommendations, we will develop a standard Excel data charting form and populate it with key study characteristics (e.g., author(s), publication year, study title, geographical region [urban/rural], study design, study methods, and key themes and issues from each selected study vis-à-vis the review objectives. This will be an iterative process, involving back-and-forth data extraction and subsequent updating of the data charting form.

We will begin the data charting process with a pilot phase where two independent reviewers (FA & ON) will chart data from a random sample of 5–10 of the final selected studies. This piloting process will determine whether or not both reviewers’ independent chartings align with the review objectives, and allow for any changes to the data charting form. Any disagreements will be discussed with a more senior researcher (WM), and the data charting form will be revised accordingly.

## Results

### Stage 5: Collating summarising and reporting results

Following Levac *et al.*’s ^36^ suggestions, we will synthesise our data in three phases. Firstly, we will analyse and summarise all charted data about study characteristics using descriptive statistics. We will use thematic analysis to analyse and summarise all charted data relating to key themes and issues vis-à-vis the review objectives. We will present quantitative results using tables and/or graphs and qualitative findings using key themes. Additionally, we will triangulate quantitative and qualitative findings for example, by ensuring the latter provides context and nuances to the former.

## Discussion

We will discuss the results of the scoping review and their implications. By mapping the breadth of studies focusing on social and gender norms, and SRH among very young adolescents, the scoping review will provide a critical evidence base to inform the development and implementation of culturally relevant interventions and further research to help improve adolescents’ SRH outcomes in SSA. Specifically, review findings will inform the co-development and evaluation of a peer-delivered gender-transformative intervention for in- and out-of-school very young adolescents to promote positive masculinity and sexual health in Zimbabwe.

By exploring what interventions have been effective, the review could be useful in conceptualising the intervention. However, as the GEAS has observed that intervention effects differ by context, and results can be highly contextual, even for settings generally considered ‘similar’ ^30^ an important consideration will be how to tailor the intervention to suit the Zimbabwean context.

The strength of our approach is in the application of a recognised, thorough and transparent approach ^32^ to review the literature and report our results. However, given a significant part of SSA is Francophone or Lusophone, and programmes in these regions are disproportionately funded, this is likely to skew study results. Additionally, including only articles published in English (as we do not have resources to analyse studies in other languages) is another potential limitation of our review.

We will share review results with diverse stakeholders through various channels including peer-reviewed publications, conferences, stakeholder meetings with organisations working with adolescents (including VYAs) and other partners implementing programmes that seek to address SRH outcomes among adolescents in SSA. We aim to inform and influence future interventions and evaluations in this area.

## Conclusion

Here, we present a protocol to scope existing research to understand social and gender norms and their influence on very young adolescents’ SRH in SSA. The identified issues and their contextual factors will help us and others develop more context-specific, bespoke interventions.

## Ethics and consent

Ethical approval and consent were not required.

## Data Availability

No data are associated with this article. Preferred Reporting Items for Systematic Reviews and Meta-Analyses Protocols (PRISMA-P) checklist is available on Open Science Framework repository. Open Science Framework: PRISMA-P checklist for How do social and gender norms influence the sexual and reproductive health of younger adolescents in sub-Saharan Africa? A scoping review **DOI** – 10.17605/OSF.IO/EW7S5. Data are available under the terms of the Creative Commons Zero "No rights reserved" data waiver (CC0 1.0 Public domain dedication).
