# Supplementary material for: How do social norms influence the sexual and reproductive health of very young adolescents in sub-Saharan Africa? A scoping review protocol
Source: Wellcome Open Res. 2025 Feb 28;9:670. Originally published 2024 Nov 12. [Version 2] doi: 10.12688/wellcomeopenres.23139.2 (PMC11894366; doi:10.12688/wellcomeopenres.23139.2)
Supplement: Peer review history

# Reviewer report: Reviewer response for version 2

**DOI:** 10.21956/wellcomeopenres.26281.r119757

**Published:** 2025-03-10

**Authors:** Erica Sedlander

**Affiliations:** 1 University of California San Francisco, San Francisco, California, USA

The authors have adequately incorporated my suggestions.

Is the study design appropriate for the research question?

Yes

Is the rationale for, and objectives of, the study clearly described?

Yes

Are sufficient details of the methods provided to allow replication by others?

Yes

Are the datasets clearly presented in a useable and accessible format?

Yes

Reviewer Expertise:

social norms, gender norms, fertility/infertility, sexual and reproductive health

I confirm that I have read this submission and believe that I have an appropriate level of expertise to confirm that it is of an acceptable scientific standard.

# Reviewer report: Reviewer response for version 2

**DOI:** 10.21956/wellcomeopenres.26281.r119759

**Published:** 2025-03-10

**Authors:** Beatrice W. Maina

**Affiliations:** 1 African Population and Health Research Center, Nairobi, Nairobi County, Kenya

No further comments

Is the study design appropriate for the research question?

Partly

Is the rationale for, and objectives of, the study clearly described?

Partly

Are sufficient details of the methods provided to allow replication by others?

Partly

Are the datasets clearly presented in a useable and accessible format?

Not applicable

Reviewer Expertise:

Gender and adolescent sexual and reproductive health

I confirm that I have read this submission and believe that I have an appropriate level of expertise to confirm that it is of an acceptable scientific standard.

# Reviewer report: Reviewer response for version 1

**DOI:** 10.21956/wellcomeopenres.25481.r113422

**Published:** 2025-01-21

**Authors:** Jennifer Gayles

**Affiliations:** 1 Save the Children, Washington, USA

**General:**

- It seems there may be some misalignment of the RQ and what the authors hope to produce. The title indicates a very broad focus on the role of norms in adolescent SRH while the Discussion section indicates that the review intends to identify successful strategies or best practices for addressing social and gender norms that influence ASRH in SSA. Understanding how social norms influence VYA SRH is not sufficient to “inform the co-development and evaluation of a peer-delivered, gender-transformative intervention for in and out-of-school younger adolescents”; that would require a different RQ focused on successful interventions. See my further comments on this in the Methods section.
- I noted that VYA is a keyword, but the paper consistently uses the term “younger adolescents”. Consider aligning with WHO language (i.e. using VYAs instead of younger adolescents).
- WHO is currently coordinating a global research prioritization exercise using CHNRI method) to propose future research topics related to VYA SRHR. They have facilitated an inclusive process drawing on an Expert Reference Group, Youth Advisory Council and public survey (online) to develop questions. Analysis is nearly complete and will likely be highly relevant to this work. Keep an eye out for results!

**Introduction:**

- Antenatal syphilis screening is an odd choice of key SRH indicator, especially for this age group. Consider instead reporting contraceptive use, antenatal visits/adherence or HIV testing, which are more widely used in comparing SRH across regions.
- This section could benefit from more background on prevalent social and gender norms in SSA beyond just the patriarchal norms discussed in paragraph 3. There are many intertwined norms related to virginity, control of female sexuality, fertility and childbearing, and FP use that are highly relevant to this review. A brief discussion (one paragraph) on these norms and how they can impede healthy SRH behaviors and service seeking would nicely set up the final paragraph of this section.
- Last paragraph: Lack of data is certainly one reason research on VYA is light, but authors might also mention other key reasons:
  - Early adolescence is generally a time of good health, when children are not as vulnerable to illness as when they are U5 or school aged.
  - (Mistaken) beliefs that VYA are too young to be provided with information on SRH and/or that they are not sexually active, which serve as obstacles to both research and program development. Integrating these concepts would strengthen the paragraph.

**Methods:**

- The question “What gaps exist in interventions relating to social and gender norms and SRH of younger adolescents in SSA?” is not clear. Do you intend to identify what kind of interventions have been implemented for VYA SRH in SSA -OR- the strengths and weakness of those interventions? Consider revising to  “What interventions relating to social and gender norms and SRH of younger adolescents in SSA have been effective?”
- Measurement of norms is still a relatively nascent field and many studies that purport to measure norms in reality measure attitudes or perceptions of norms. This may influence the quality of studies you identify; it would be helpful to take this into account in your inclusion/exclusion criteria.
- Given how much of SSA is Francophone or Lusophone, and the fact that Western donors disproportionately fund research and programs in non-Anglophone priority countries, this is a major limitation. It may skew your results heavily.

**Useful References:**

1. Igras S, Maciera M, Murphy E, Lundgren R. Investing in very young adolescents' sexual and reproductive health. (2014) – This was a seminal piece in the VYA SRH literature and accelerated attention and resourcing for VYAs in the 2010s. DOI: 10.1080/17441692.2014.908230
2. Mmari K, Simon C, Verna R. Gender-Transformative Interventions for Young Adolescents: What Have We Learned and Where Should We Go? (2024) https://doi.org/10.1016/j.jadohealth.2024.04.016 - Publication based on a landscape review conducted in 2020: https://resourcecentre.savethechildren.net/document/very-young-adolescent-sexual-and-reproductive-health-landscape-where-are-we-now-where-do-we/
3. Passages Project published numerous studies on norms-focused programs for VYA in Mali and DRC: https://www.irh.org/projects/passages/. Publications and grey literature are (mostly) in English. Results of an SRH-focused social norms exploration among VYAs in Kinshasa may be of particular interest, though it is in French: https://www.growingupgreat.org/resources/

Is the study design appropriate for the research question?

Yes

Is the rationale for, and objectives of, the study clearly described?

Partly

Are sufficient details of the methods provided to allow replication by others?

Yes

Are the datasets clearly presented in a useable and accessible format?

Not applicable

Reviewer Expertise:

social and gender norms; adolescent sexual and reproductive health with focus on very young adolescents; design, pilot and scale up of programs

I confirm that I have read this submission and believe that I have an appropriate level of expertise to confirm that it is of an acceptable scientific standard, however I have significant reservations, as outlined above.

## References

[1] : Investing in very young adolescents' sexual and reproductive health. *Glob Public Health* .2014;9(5) : 10.1080/17441692.2014.908230 555-69 10.1080/17441692.2014.908230 24824757 PMC4066908

[2] : Gender-Transformative Interventions for Young Adolescents: What Have We Learned and Where Should We Go?. *J Adolesc Health* .2024;75(4S) : 10.1016/j.jadohealth.2024.04.016 S62-S80 10.1016/j.jadohealth.2024.04.016 39293879

[3] Passages Project published numerous studies on norms-focused programs for VYA in Mali and DRC: https://www.irh.org/projects/passages/. Publications and grey literature are (mostly) in English. Results of an SRH-focused social norms exploration among VYAs in Kinshasa may be of particular interest, though it is in French: https://www.growingupgreat.org/resources/.

# Response

**Published:** 2025-02-16

**Authors:** Owen Nyamwanza

**Affiliations:** SRH & MNCH, Centre for Sexual Health and HIV/AIDS Research Zimbabwe, Harare, Harare Province, Zimbabwe

**Comments:**

**General:**

- It seems there may be some misalignment of the RQ and what the authors hope to produce. The title indicates a very broad focus on the role of norms in adolescent SRH while the Discussion section indicates that the review intends to identify successful strategies or best practices for addressing social and gender norms that influence ASRH in SSA. Understanding how social norms influence VYA SRH is not sufficient to “inform the co-development and evaluation of a peer-delivered, gender-transformative intervention for in and out-of-school younger adolescents”; that would require a different RQ focused on successful interventions. See my further comments on this in the Methods section.

**  Response:** Thank you. Based on this and other reviewers’ suggestions we have revised the sub-questions to: *“What interventions relating to social and gender norms and SRH of younger adolescents in SSA have been effective?”* (lines 137-138).  

**Comments:** 

- I noted that VYA is a keyword, but the paper consistently uses the term “younger adolescents”. Consider aligning with WHO language (i.e. using VYAs instead of younger adolescents).
**Response:** Thank you. We now use VYA throughout to align with WHO language.  

**Comments:**

- WHO is currently coordinating a global research prioritization exercise using CHNRI method) to propose future research topics related to VYA SRHR. They have facilitated an inclusive process drawing on an Expert Reference Group, Youth Advisory Council and public survey (online) to develop questions. Analysis is nearly complete and will likely be highly relevant to this work. Keep an eye out for results!

**Response: **Thank you for the heads-up.  

**Comments:**

**Introduction:**

- Antenatal syphilis screening is an odd choice of key SRH indicator, especially for this age group. Consider instead reporting contraceptive use, antenatal visits/adherence or HIV testing, which are more widely used in comparing SRH across regions.

**Response:** Thank you. We now cite these indicators (lines 76-78).  

**Comments:**

- This section could benefit from more background on prevalent social and gender norms in SSA beyond just the patriarchal norms discussed in paragraph 3. There are many intertwined norms related to virginity, control of female sexuality, fertility and childbearing, and FP use that are highly relevant to this review. A brief discussion (one paragraph) on these norms and how they can impede healthy SRH behaviors and service seeking would nicely set up the final paragraph of this section.

**Response: **Thank you. We now include a paragraph on these norms (lines 92-96).  

**Comments:**

- Last paragraph: Lack of data is certainly one reason research on VYA is light, but authors might also mention other key reasons:
  - Early adolescence is generally a time of good health, when children are not as vulnerable to illness as when they are U5 or school aged.
  - (Mistaken) beliefs that VYA are too young to be provided with information on SRH and/or that they are not sexually active, which serve as obstacles to both research and program development. Integrating these concepts would strengthen the paragraph.

**Response:** Thank you. We now include a paragraph on these key reasons (lines 108-112).

**Comments:**

**Methods:**

- The question “What gaps exist in interventions relating to social and gender norms and SRH of younger adolescents in SSA?” is not clear. Do you intend to identify what kind of interventions have been implemented for VYA SRH in SSA -OR- the strengths and weakness of those interventions? Consider revising to  “What interventions relating to social and gender norms and SRH of younger adolescents in SSA have been effective?”

**Response:** Thank you for this suggestion. We have revised the sub-question as suggested (lines 137- 138).  

**Comments:**

- Measurement of norms is still a relatively nascent field and many studies that purport to measure norms in reality measure attitudes or perceptions of norms. This may influence the quality of studies you identify; it would be helpful to take this into account in your inclusion/exclusion criteria.

**Response:** Thank you. We now include attitudes or perceptions of norms in the search strategy (Table 1) and inclusion/exclusion criteria (Table 2).  

**Comments:**

- Given how much of SSA is Francophone or Lusophone, and the fact that Western donors disproportionately fund research and programs in non-Anglophone priority countries, this is a major limitation. It may skew your results heavily.

**Response:** Thank you. We now include this limitation (lines 225-227).  

**Comments:**

**Useful References:**

1. Igras S, Maciera M, Murphy E, Lundgren R. Investing in very young adolescents' sexual and reproductive health. (2014) – This was a seminal piece in the VYA SRH literature and accelerated attention and resourcing for VYAs in the 2010s. DOI: 10.1080/17441692.2014.908230
2. Mmari K, Simon C, Verna R. Gender-Transformative Interventions for Young Adolescents: What Have We Learned and Where Should We Go? (2024) https://doi.org/10.1016/j.jadohealth.2024.04.016 - Publication based on a landscape review conducted in 2020: https://resourcecentre.savethechildren.net/document/very-young-adolescent-sexual-and-reproductive-health-landscape-where-are-we-now-where-do-we/
3. Passages Project published numerous studies on norms-focused programs for VYA in Mali and DRC: https://www.irh.org/projects/passages/. Publications and grey literature are (mostly) in English. Results of an SRH-focused social norms exploration among VYAs in Kinshasa may be of particular interest, though it is in French: https://www.growingupgreat.org/resources/

**  Response:** Thank you for the references. We have incorporated some of these.

# Reviewer report: Reviewer response for version 1

**DOI:** 10.21956/wellcomeopenres.25481.r115191

**Published:** 2024-12-23

**Authors:** Beatrice W. Maina

**Affiliations:** 1 African Population and Health Research Center, Nairobi, Nairobi County, Kenya

**Title**: Needs clarity on whether the focus is gender norms or social norms. Social norms encompasses gender norms, hence it gives a broader perspective. The PCC inclusion criteria also refers to social norms.

**Abstract**: the abstract needs to be strengthened. The "PRISMA Extension for Scoping Reviews (PRISMA-ScR): Checklist and Explanation" might be useful. 

 The objective of the intended scoping review is not clear. The authors argue there is limited evidence among younger adolescents, which is true, as there are studies that have shown limited but growing evidence on social norms (and the sub-components) among younger adolescents. The only difference maybe the time period and therefore need to articulate what new knowledge the review is bringing forth.

**Introduction**

 The article "Sawyer, Susan M et al. The age of adolescence. The Lancet Child & Adolescent Health, Volume 2, Issue 3, 223 - 228" might be useful when defining the broad adolescence age-range and the sub-groups therein

**Methods**

 The second research question brings in an intervention angle nor clearly mentioned prior. This may also have implications on the framework used. Perhaps PICO.

 In the PCC framework as well as the search terms, there is nowhere gender is mentioned as a concept.

 Authors could also consider other concepts such as "teenage", "adolescent", "pregnancy" etc.

 Under study selection, the authors indicate they will include studies that meet at least one of the criteria. This may create a pool of studies that are not within the current scope of the review.

 Will the searches for the two research questions be combined or will they be conducted separately? And what is research questions 2 exactly aims to answer? For instance research gaps in what?

 On quality appraisal, might be good to mention some examples of appraisal tools to be used, and also an indication on whether any studies will be dropped if they do not meet a certain threshold.

**Results**

 Will be important to add how quantitative and qualitative findings will be triangulated to inform key findings and discussions. Additionally, how will the results form the two research questions will be presented needs to be outlined.

**Discussion**

 The discussion section seems to capture the goal of the study "mapping the breadth of studies focusing on social and gender norms, and SRH among younger adolescents" which is more clearer than the objective stated earlier.

 The discussion should focus on the results of the scoping review. The discussion is envisioned to respond to a specific need, that is, to inform an intervention in Zimbabwe for in- and out- of school younger adolescents. This information is presented abruptly in the discussion. While the review could be useful in conceptualizing the intervention, noting the diversity of norms in the continent, there maybe need for a more contextualized focus on Zimbabwe.  

**Conclusion**

 May need to highlight the strengths and limitations of the study

Is the study design appropriate for the research question?

Partly

Is the rationale for, and objectives of, the study clearly described?

Partly

Are sufficient details of the methods provided to allow replication by others?

Partly

Are the datasets clearly presented in a useable and accessible format?

Not applicable

Reviewer Expertise:

Gender and adolescent sexual and reproductive health

I confirm that I have read this submission and believe that I have an appropriate level of expertise to confirm that it is of an acceptable scientific standard, however I have significant reservations, as outlined above.

## References

[1] The age of adolescence Sawyer, Susan M et al. The Lancet Child & Adolescent Health, Volume 2, Issue 3, 223 - 228.

# Response

**Published:** 2025-02-16

**Authors:** Owen Nyamwanza

**Affiliations:** SRH & MNCH, Centre for Sexual Health and HIV/AIDS Research Zimbabwe, Harare, Harare Province, Zimbabwe

**Comments:**

**Title**: Needs clarity on whether the focus is gender norms or social norms. Social norms encompasses gender norms; hence it gives a broader perspective. The PCC inclusion criteria also refers to social norms.

**Response:** Thank you. Based on this and the other reviewer’s comment, we now just use “social norms” in the title.

**Comments:**

**Abstract**: the abstract needs to be strengthened. The "PRISMA Extension for Scoping Reviews (PRISMA-ScR): Checklist and Explanation" might be useful. 

**Response:** Thank you. We have strengthened the abstract but keeping in line with the word limit. Also, we will report findings in accordance with the guidance provided in the Preferred Reporting Items for Systematic Reviews and Meta-Analyses Protocols (PRISMA-P) statement (lines 36-37).

**Comments:**

 The objective of the intended scoping review is not clear. The authors argue there is limited evidence among younger adolescents, which is true, as there are studies that have shown limited but growing evidence on social norms (and the sub-components) among younger adolescents. The only difference maybe the time period and therefore need to articulate what new knowledge the review is bringing forth.

**Response:** Thank you. Based on this and the other reviewers’ comments, we now state how the Global Early Adolescent Study has observed that intervention effects do differ by context, and results can be highly contextual, even for settings generally considered ‘similar’. We also state that there is increased recognition that African masculinities are produced in unique and varying contexts of intersections (including class, ethnicity, sexuality), which points to the need for context-specific, bespoke initiatives. We then state that the proposed scoping review seeks to identify and analyse literature on how social and gender norms influence younger adolescents’ SRH in SSA and that the identified issues and their contextual factors will help us develop a context-specific, bespoke intervention (lines 112-127).

**Comments:**

**Introduction**

 The article "Sawyer, Susan M et al. The age of adolescence. The Lancet Child & Adolescent Health, Volume 2, Issue 3, 223 - 228" might be useful when defining the broad adolescence age-range and the sub-groups therein.

**Response:** Thank you for pointing us to this article. We now sate that, “Exactly when adolescence starts and ends has been a subject of debate, especially as some view a 10-year-old as a child and a 19-year-old as an adult [3]. We are guided by the World Health Organisation (WHO) which defines “adolescents” as individuals in the 10-19 years age group, “youth” as those in the 15-24 years range, while “young people” covers those 10-24 years old [4] (lines 60-63).

**Comments:**

**Methods**

 The second research question brings in an intervention angle nor clearly mentioned prior. This may also have implications on the framework used. Perhaps PICO.

**Response: **Based on this and other reviewers’ suggestions we have revised the sub-questions to: *“What interventions relating to social and gender norms and SRH of younger adolescents in SSA have been effective?”* (lines 137-138).

**Comments:**

 In the PCC framework as well as the search terms, there is nowhere gender is mentioned as a concept.

**Response: **Thank you. We already mention “gender norms” in the PCC framework (Table 2). We now include “gender norms” as a concept and include search terms (Table 1).  

**Comments: **Authors could also consider other concepts such as "teenage", "adolescent", "pregnancy" etc.

**Response: **Thank you for these very helpful suggestions. We have added these to the search terms (Table 1).

**Comments: **Under study selection, the authors indicate they will include studies that meet at least one of the criteria. This may create a pool of studies that are not within the current scope of the review.

**Response:** Thank you. We have revised this section (lines 169-172).

**Comments:**

 Will the searches for the two research questions be combined or will they be conducted separately? And what is research questions 2 exactly aims to answer? For instance research gaps in what?

**Response:** The searches for the two research questions will be combined. Based on this and other reviewers’ suggestions we have revised the sub-questions to: *“What interventions relating to social and gender norms and SRH of younger adolescents in SSA have been effective?”* (lines 137-138).  

**Comments:**

 On quality appraisal, might be good to mention some examples of appraisal tools to be used, and also an indication on whether any studies will be dropped if they do not meet a certain threshold.

**Response: **For a separate scoping review, reviewers have discouraged us from conducting a quality appraisal as this is not usually done in scoping reviews. We therefore no longer mention “Quality appraisal’ here (but stand guided).  

**Comments:**

**Results**

 Will be important to add how quantitative and qualitative findings will be triangulated to inform key findings and discussions.

**Response:** Thank you. We now mention this (lines 207-208).  

**Comments:**

 Additionally, how the results from the two research questions will be presented needs to be outlined.

**Response:** We now outline this (lines 203-207).  

**Comments:**

**Discussion**

 The discussion section seems to capture the goal of the study "mapping the breadth of studies focusing on social and gender norms, and SRH among younger adolescents" which is clearer than the objective stated earlier.

**Response: **Thank you. We have now revised the study goal to reflect this (lines 125-127).

**Comments:**

 The discussion should focus on the results of the scoping review.

**Response: **We now state that the Discussion will discuss the results (line 211).  

**Comments:**

 The discussion is envisioned to respond to a specific need, that is, to inform an intervention in Zimbabwe for in- and out- of school younger adolescents. This information is presented abruptly in the discussion. While the review could be useful in conceptualizing the intervention, noting the diversity of norms in the continent, there may be need for a more contextualized focus on Zimbabwe.  

**Response:** Thank you. We now sate that, “By exploring what interventions have been effective, the review could be useful in conceptualising the intervention. However, as the GEAS has observed that intervention effects differ by context, and results can be highly contextual, even for settings generally considered ‘similar’ [34] an important consideration will be how to tailor the intervention to suit the Zimbabwean context (lines 219-222).  

**Conclusion**

 May need to highlight the strengths and limitations of the study.

**Response: **Thank you. We now include these (lines 224-229).  

**References** The age of adolescence Sawyer, Susan M et al. The Lancet Child & Adolescent Health, Volume 2, Issue 3, 223 - 228.

**Response: **Thank you for the reference. It has helped us discuss the age of adolescence (lines 60-63).

# Reviewer report: Reviewer response for version 1

**DOI:** 10.21956/wellcomeopenres.25481.r111447

**Published:** 2024-12-10

**Authors:** Erica Sedlander

**Affiliations:** 1 University of California San Francisco, San Francisco, California, USA

**Summary:** The authors present a clear, succinct protocol for a scoping review protocol on how social and gender norms influence the sexual and reproductive health of younger adolescents in SSA.

**Major comments:**

 My main comment is that the protocol is quite broad in that it argues that there is a gap around how social norms affect younger adolescents specifically. However, authors do not cite all the existing work. They only state there is a gap in the literature on social norms among young adolescents. The Global Early Adolescent Study is one example of a huge effort focused on this topic specifically. https://www.geastudy.org/.

 Additionally, the reason for conducting this review is that adolescents are ignored but then in the discussion, they go into a very specific intervention. Perhaps more specific reasons than just a lack of literature is warranted.

**Minor comments:  **

- **Title**: Gender norms are a subset of social norms so you can just use “social norms” in the title

**Abstract**

- Sentence 1: “Younger adolescents (aged 10–14 years) in sub-Saharan Africa (SSA) have disproportionate sexual and reproductive health (SRH) outcomes due to structural, behavioural, socioeconomic and other factors.
- I believe they have unique needs not more outcomes.

**Discussion**

- The review will generate the most up-to-date evidence and identify gaps in THE literature in addition to informing future research on how gender and social norms influence younger adolescents’ SRH in SSA. 
- VAGUE: Findings will inform and influence future interventions and evaluations in this area. You have a more specific goal listed in the discussion.

**Introduction**

- What’s the age range of what’s considered adolescents in SSA?
- Suggested edit: “75% of NEW HIV infections globally.”
  |
- Too much of a jump to social norms paragraph (need a transition).
- “Resultantly, there is a paucity of research on the influence of gender and social norms on younger adolescents’ SRH.” The DHS doesn’t collect data on social or gender norms specifically. They collect data on individual attitudes and beliefs which is different from norms.

**Methods**

- “We do not have resources to analyze studies written in other languages, and we recognise that this is a potential limitation of our review.” Save for a limitations section.
- ***Quality appraisal*.** We will assess quality using relevant tools and approaches depending on a study’s design and methodology. There are specific quality appraisal tools like MMAT mixed methods appraisal tool.
- “Any disagreements will be discussed with an expert (WM).” Is WM an expert in scoping reviews? 

**Results**

- How will you present qual versus quant results?

**Discussion**

- Suggest removing the word “current. ”Knowledge around influence of social and gender norms on SSA’s younger adolescents’ SRH is currently limited ^10^ . This scoping review will synthesise current literature to address this critical knowledge gap.”
- In the discussion you state, “Specifically, review findings will inform the co-development and evaluation of a peer-delivered gender-transformative intervention for in- and out-of-school younger adolescents to promote positive masculinity and sexual health in Zimbabwe. The ultimate goal is to develop an effective model for scale-up to influence constructs of positive masculinity.” However, the abstract is much more vague and feels broad. Perhaps grounding in it why you need this information for your study will infuse it with more concrete examples of what is missing and how that will inform this peer-led intervention. Since you are missing existing data on young adolescents, its hard to be specific about the exact gaps. You just state a dearth without telling us what you need to know and how that will inform your intervention or anyone’s intervention.

**Conclusion**

- “The review will provide valuable insights for the development of tailored interventions for this important group.” This is vague and not that helpful. A brief review of existing literature which illuminates the gaps will show what this review could provide for your intervention and others.

Is the study design appropriate for the research question?

Yes

Is the rationale for, and objectives of, the study clearly described?

Yes

Are sufficient details of the methods provided to allow replication by others?

Yes

Are the datasets clearly presented in a useable and accessible format?

Yes

Reviewer Expertise:

social norms, gender norms, fertility/infertility, sexual and reproductive health

I confirm that I have read this submission and believe that I have an appropriate level of expertise to confirm that it is of an acceptable scientific standard, however I have significant reservations, as outlined above.

## References

[1] *Global Early Adolescent Study* . Reference source

# Response

**Published:** 2025-02-16

**Authors:** Owen Nyamwanza

**Affiliations:** SRH & MNCH, Centre for Sexual Health and HIV/AIDS Research Zimbabwe, Harare, Harare Province, Zimbabwe

**Comments:**

**Summary:** The authors present a clear, succinct protocol for a scoping review protocol on how social and gender norms influence the sexual and reproductive health of younger adolescents in SSA.

**Response: **Thank you for noting that this is a clear, succinct protocol.

**Major comments:**

 My main comment is that the protocol is quite broad in that it argues that there is a gap around how social norms affect younger adolescents specifically. However, authors do not cite all the existing work. They only state there is a gap in the literature on social norms among young adolescents. The Global Early Adolescent Study is one example of a huge effort focused on this topic specifically. https://www.geastudy.org/.

 Additionally, the reason for conducting this review is that adolescents are ignored but then in the discussion, they go into a very specific intervention. Perhaps more specific reasons than just a lack of literature is warranted.

**Response: **Thank you for highlighting The Global Early Adolescent Study.   We now state how the Global Early Adolescent Study has observed that intervention effects do differ by context, and results can be highly contextual, even for settings generally considered ‘similar’. We also state that there is increased recognition that African masculinities are produced in unique and varying contexts of intersections (including class, ethnicity, sexuality), which points to the need for context-specific, bespoke initiatives. We then state that the proposed scoping review seeks to map the breadth of studies focusing on social and gender norms, and SRH among VYAs, in addition to exploring interventions that have been effective. Findings will inform the development of a context-specific, bespoke intervention (lines 112-127).  

**Minor comments:  **

- **Title**: Gender norms are a subset of social norms so you can just use “social norms” in the title

**Response: **Thank you. We now just use “social norms” in the title.  

**Comments:**

**Abstract**

- Sentence 1: “Younger adolescents (aged 10–14 years) in sub-Saharan Africa (SSA) have disproportionate sexual and reproductive health (SRH) outcomes due to structural, behavioural, socioeconomic and other factors.
   I believe they have unique needs not more outcomes.

**Response: **We have revised the sentence to reflect that they have the worst SRH outcomes of this age group worldwide. Later, we provide examples (lines 73-80).

**Comments:**

**Discussion**

- The review will generate the most up-to-date evidence and identify gaps in THE literature in addition to informing future research on how gender and social norms influence younger adolescents’ SRH in SSA. 
  VAGUE: Findings will inform and influence future interventions and evaluations in this area. You have a more specific goal listed in the discussion.

**Response:** We have revised to: “The review will map the breadth of studies focusing on social and gender norms, and SRH among VYAs, in addition to exploring interventions that have been effective. Findings will help us and others develop context-specific, bespoke interventions” (lines 43-45).

**Comments:**

**Introduction**

- What’s the age range of what’s considered adolescents in SSA?

**Response:** We now state that these are 10-19 years old (line 65) but state that “Exactly when adolescence starts and ends has been a subject of debate, especially as some view a 10-year-old as a child and a 19-year-old as an adult [3]. We are guided by the World Health Organisation (WHO) which defines “adolescents” as individuals in the 10-19 years age group, “youth” as those in the 15-24 years range, while “young people” covers those 10-24 years old [4] (lines 60-63).

**Comments:**

 Suggested edit: “75% of NEW HIV infections globally.”

**Response: **We have revised as suggested (line 79).

**Comments:**

 Too much of a jump to social norms paragraph (need a transition).

**Response: **Thank you. We now introduce social norms (line 80).  

**Comments:**

 “Resultantly, there is a paucity of research on the influence of gender and social norms on younger adolescents’ SRH.” The DHS doesn’t collect data on social or gender norms specifically. They collect data on individual attitudes and beliefs which is different from norms.

**Response: **We agree. Based on this and another reviewer’s comment, we have rewritten the paragraph (lines 92-96).  

**Comments:**

**Methods**

- “We do not have resources to analyse studies written in other languages, and we recognise that this is a potential limitation of our review.” Save for a limitations section. 

**Response:** Thank you. We now include this as a limitation (lines 227-229).  

**Comments:**

- ***Quality appraisal*** **.** We will assess quality using relevant tools and approaches depending on a study’s design and methodology. There are specific quality appraisal tools like MMAT mixed methods appraisal tool.

**Response:** For a separate scoping review, reviewers have discouraged us from conducting a quality appraisal as this is not usually done in scoping reviews. We therefore no longer mention “Quality appraisal’ (but stand guided).  

**Comments:**

- “Any disagreements will be discussed with an expert (WM).” Is WM an expert in scoping reviews? 

**Response:** We now say, ‘a more senior researcher’ (line 198).

**Comments:**

**Results**

- How will you present qual versus quant results?

**Response: **“We will present quantitative results using tables and/or graphs and qualitative findings using key themes. Additionally, we will triangulate quantitative and qualitative findings for example, by ensuring the latter provides context and nuances to the former” (lines 205-208).  

**Comments:**

**Discussion**

- Suggest removing the word “current. ”Knowledge around influence of social and gender norms on SSA’s younger adolescents’ SRH is currently limited ^10^ . This scoping review will synthesise current literature to address this critical knowledge gap.” 

**Response:** Thank you. We have removed as suggested.  

**Comments:**

- In the discussion you state, “Specifically, review findings will inform the co-development and evaluation of a peer-delivered gender-transformative intervention for in- and out-of-school younger adolescents to promote positive masculinity and sexual health in Zimbabwe. The ultimate goal is to develop an effective model for scale-up to influence constructs of positive masculinity.” However, the abstract is much vaguer and feels broad. Perhaps grounding in it why you need this information for your study will infuse it with more concrete examples of what is missing and how that will inform this peer-led intervention. Since you are missing existing data on young adolescents, it’s hard to be specific about the exact gaps. You just state a dearth without telling us what you need to know and how that will inform your intervention or anyone’s intervention.

**Response:** Thank you. As stated above, we now state how the Global Early Adolescent Study has observed that intervention effects do differ by context, and results can be highly contextual, even for settings generally considered ‘similar’. We also state that there is increased recognition that African masculinities are produced in unique and varying contexts of intersections (including class, ethnicity, sexuality), which points to the need for context-specific, bespoke initiatives. We then state that the proposed scoping review seeks to identify and analyse literature on how social and gender norms influence younger adolescents’ SRH in SSA, information which is essential for the development of bespoke interventions (lines 112-127).

**Comments:**

**Conclusion**

- “The review will provide valuable insights for the development of tailored interventions for this important group.” This is vague and not that helpful. A brief review of existing literature which illuminates the gaps will show what this review could provide for your intervention and others.

**Response: **Thank you. We have revised the conclusion to highlight how the proposed review will help us and others develop more context-specific, bespoke interventions (lines 238-239).
